# Dataset for an analysis of communicative aspects of finance

**DOI:** 10.1016/j.dib.2017.01.012

**Published:** 2017-02-09

**Authors:** Natalya Zavyalova

**Affiliations:** Ural Federal University, 19 Mira Street, Ekaterinburg 620002, Russia

**Keywords:** Communication, Information retrieval, Semantic similarity measure, Finance

## Abstract

The article describes a step-by-step strategy for designing a universal comprehensive vision of a vast majority of financial research topics. The strategy is focused around the analysis of the retrieval results of the word processing system Serelex which is based on the semantic similarity measure. While designing a research topic, scientists usually employ their individual background. They rely in most cases on their individual assumptions and hypotheses. The strategy, introduced in the article, highlights the method of identifying components of semantic maps which can lead to a better coverage of any scientific topic under analysis. On the example of the research field of finance we show the practical and theoretical value of semantic similarity measurements, i.e., a better coverage of the problems which might be included in the scientific analysis of financial field. At the designing stage of any research scientists are not immune to an insufficient and, thus, erroneous spectrum of problems under analysis. According to the famous maxima of St. Augustine, ‘Fallor ergo sum’, the researchers’ activities are driven along the way from one mistake to another. However, this might not be the case for the 21st century science approach. Our strategy offers an innovative methodology, according to which the number of mistakes at the initial stage of any research may be significantly reduced. The data, obtained, was used in two articles (N. Zavyalova, 2017) [7], (N. Zavyalova, 2015) [8]. The second stage of our experiment was driven towards analyzing the correlation between the language and income level of the respondents. The article contains the information about data processing.

**Specifications Table**

**Table:** 

**Data set 1**	
Subject area	Cognitive Psychology
More specific subject area	Linguistics and communication
Type of data	2 graphs
How data was acquired	With the help of Serelex system
Data format	**.jpeg**
**Experimental factors**	**words**
**Experimental features**	The data helps see the communicative aspects of finance better and helps communicate finance more efficiently, according to a certain brain map.
	The information is relevant for experts working in the sphere of financial journalism, blogging and mass media.
Data source location	http://serelex.cental.be/#financehttp://serelex.cental.be/#money
	http://serelex.cental.be/ru#финансы

**Data set 2**

**Table:** 

Subject area	Psycholinguistics
More specific subject area	Linguistics and communication
Type of data	2 tables
How data was acquired	Levada Centre omnibus survey
Data format	**numbers**
**Experimental factors**	**opinions**
**Experimental features**	The data helps understand the correlation between the attitude to words and the income level of the respondents
Data source location	Levada Centre, RF, Moscow

**Value of the data**

This data helps enrich the knowledge in the following research areas of finance:

• The correlation of the head word (‘‘money’’, ‘‘finance’’) and satellite subjects. This correlation is relevant because it helps see if all the subjects are included in the research design, what additional directions may be implemented for a more complete research panorama.
• Is there any correlation between income levels and the attitude to the language people speak?
• Should we be more attentive to the language we use?
• Can a language be an indirect determinant of the income level?
• What money topics should you talk about to get your audience the most interested?
• Which of your existing money posts should you share today for more traffic? More leads?
• Which post should you revise and enrich with financial details for a better conversion rate?

If you include the head items from the graph in your research interests and collecting facts, you׳ll see how the world of finance functions in terms of mental structures reflected in the English language. Mental activities are widely discussed at the level of brain activity [1]. However, we see a potential to analyze mental activity at the level of words. It is possible to conduct a cross-cultural analysis of the ‘money’ and ‘finance’ concepts in English and in Russian. This can bring you to a better understanding of cultural difference of financial issues and new cross-cultural research fields. These issues are of much importance in connection with tourism and finance in general [2].

The next step of understanding the communicative aspect of finance is through the attitude of people with different income levels towards their day-to-day language. Here we provide the results of 2 consecutive omnibus surveys (2014, 2016), describing the correlation between the attitude to popular words and income. Our central hypothesis was that people with higher income levels are more attentive to the language they use.

## 1 Data

The first data set is given in the format of two graphs with the head word ‘‘money’’ and ‘‘finance’’. The nodes are connected with the words which are closely related to the head words in the Internet. In case you suffer from a shortage of topics for research of money and finance, you can use the node-words for signposts, directing you to a new research field. This data presents a form of a mind map providing reliable clues for further research. You can use this data for collecting facts about one language or several languages in comparison [4].

.

The next step of our financial research was an attempt to see, if there any correlation between the income level and the attitude to the language people use. The second data set is given in the form of omnibus survey results. The respondents had to describe their income levels, according to the goods they could buy (‘‘only food’’, ‘‘food and clothes’’, ‘‘automobile’’) answering the following question, “do you often use idioms and phrases in your day-to-day conversations?”. And then their answers about their income levels were compared with their answers to the main question of the survey.

.

## 2 Experimental design, materials and methods

The experimental design of our research was based on the assumption that a better semantic coverage of money issues can lead us to new fields of further research. Although the retrieval of such notions as ‘credit card’, ‘banking’, ‘interest rate’, ‘asset’ was predictable, the inclusion of such notions as ‘information’ and ‘telecommunication’ led us to the conclusion that in our research we might specify certain communicative areas of financial policies of the present and the future. Thus, we decided to conduct a research of communicative policy of the NDB (BRICS Development Bank) [6].

The developers of Serelex system describe the design stage of their system the following way, ‘‘We extended a set of the 6 classical Hearst [3] patterns (1–6) with 12 further patterns (7–18), which aim at extracting hypernymic and synonymic relations. The patterns are encoded in finite-state transducers (FSTs) with the help of the corpus processing tool UNITEX 1:

1. such NP as NP, NP[,] and/or NP;
2. NP such as NP, NP[,] and/or NP;
3. NP, NP [,] or other NP;
4. NP, NP [,] and other NP;
5. NP, including NP, NP [,] and/or NP;
6. NP, especially NP, NP [,] and/or NP; 1 [9]
7. NP: NP, [NP,] and/or NP;
8. NP is DET ADJ.Superl NP;
9. NP, e. g., NP, NP[,] and/or NP;
10. NP, for example, NP, NP[,] and/or NP;
11. NP, i. e.[,] NP;
12. NP (or NP);
13. NP means the same as NP;
14. NP, in other words[,] NP;
15. NP, also known as NP;
16. NP, also called NP;
17. NP alias NP;
18. NP aka NP. Patterns are based on linguistic knowledge and thus provide a more precise representation than co-occurences or bag-of-word models.

UNITEX makes it possible to build negative and positive contexts, to exclude meaningless adjectives, and so on’’ [5].

The experiment is based on the information retrieval method, offered by Serelex system. The system is easy and is a completely public domain. All you need is just type in a head word and the graph starts developing on your screen. The system is available in two languages: English and Russian.

The second stage of our research was centered around the correlation between the language and the income level of respondents. The omnibus survey results were obtained with the help of Levada Center in Russia. The survey stage was done according to the following strategy.

Levada Centre applies the data of representative sample of urban and rural population of Russia, 1600 persons aged 18 years and older.

Universe population is assumed as entire adult population of Russia excluding the following categories:

• persons, doing their military service by conscription (around 1% of total adult population);
• persons under imprisonment before trial or convicted (around 0.8% of total adult population);
• persons living in remote or difficult to access regions of Far North (around 1,9% of total adult population);
• population of Chechen Republic and Ingushetia Republic (1,1% of total adult population);
• persons, residing in rural settlements with not more than 50 inhabitants (around 0,8% of total adult population);
• persons with mental diseases, constantly living in psycho-neurological hospitals (about 1,2% of adult population).

Sample of the omnibus wass distributed among 8 federal districts(1 – North-Western, 2 – Central, 3 -Volga, 4 – Southern, 5 – North Caucassian, 6 – Ural, 7 – Siberian, and 8 – Far Eastern), and inside each district – among 5 strata of settlements proportionally to number of population living in them in age of 18+ years. All cities with over 1 mln. population were inserted in the sample as self-representative units. In the rest strata with probability, proportional to size of a settlement, there were selected from 1 to 8 urban settlements (rural districts in rural area), so that 7–13 interviews are conducted in each of them. Number of interviews, falling onto one strata, was divided equally among selected settlements. Totally there were selected for the study 130 PSUs (94 urban settlements and 36 rural districts in 45 subjects of Russian Federation) [6].

The strategy of identifying relevant features of any concept with the help of semantic similarity measure of Serelex system made it possible to cover a broader scope of financial features which led us to unexpected conclusions and resulted in a bigger research of BRICS money policy [7]. If it had not been for this system, we would have overlooked quite a number of relevant features.

The correlation between the income level and the attitude towards to the language people used resulted into the discovery that people with higher income levels are more attentive to the language they use. They are willing to analyze the language they speak and they admit the role of idioms in their day-to-day discourses [8]. Those respondents who could afford it to buy an automobile in both surveys admitted their tendency to use idioms in day-to-day discourses. Language awareness may be viewed as an indirect income determinant.
