# Supplementary material for: Dataset for an analysis of communicative aspects of finance
Source: Data Brief. 2017 Feb 9;11:197–203. doi: 10.1016/j.dib.2017.01.012 (PMC5315436; doi:10.1016/j.dib.2017.01.012)
Supplement: Supplementary file 1 — Supplementary material [file mmc1.docx]

Conflict of Interest Form

By this form I state that my paper “**DATASET FOR AN ANALYSIS OF COMMUNICATIVE ASPECTS OF FINANCE**” doesn’t contain any conflict of interest. All acknowledgements are specified.
